# Supplementary figures and images for: Effects of specialist care lower limb orthoses on personal goal attainment and walking ability in adults with neuromuscular disorders
Source: PLoS One. 2023 Jan 18;18(1):e0279292. doi: 10.1371/journal.pone.0279292 (PMC9847977; doi:10.1371/journal.pone.0279292)

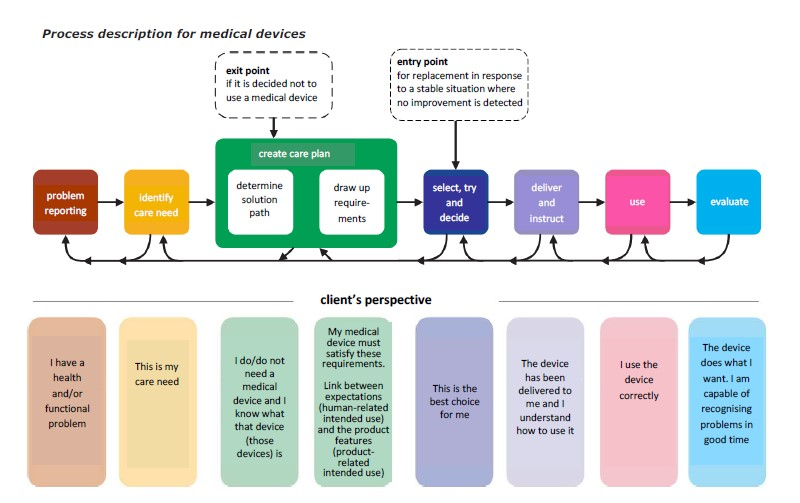

Supplement: S1 Fig — (TIF) [file pone.0279292.s001.tif]
