# Supplementary material for: Effects of specialist care lower limb orthoses on personal goal attainment and walking ability in adults with neuromuscular disorders
Source: PLoS One. 2023 Jan 18;18(1):e0279292. doi: 10.1371/journal.pone.0279292 (PMC9847977; doi:10.1371/journal.pone.0279292)
Supplement: S1 Checklist — (DOCX) [file pone.0279292.s003.docx]

STROBE Statement—checklist of items that should be included in reports of observational studies

|  | Item No. | Recommendation | Page  No. | Relevant text from manuscript |
| --- | --- | --- | --- | --- |
| **Title and abstract** | 1 | (*a*) Indicate the study’s design with a commonly used term in the title or the abstract | 2 | Chort study |
|  |  | (*b*) Provide in the abstract an informative and balanced summary of what was done and what was found | 2 | Three months after provision, the specialist care orthosis was compared to the usual care orthosis worn at baseline in terms of personal goal attainment (Goal Attainment Scaling (GAS)), comfortable walking speed (m/s), net energy cost (J/kg/m) (both assessed during a 6-minute walk test), perceived walking ability and satisfaction |
| Introduction | | | |  |
| Background/rationale | 2 | Explain the scientific background and rationale for the investigation being reported | 4-5 |  |
| Objectives | 3 | State specific objectives, including any prespecified hypotheses | 5 | In this study, we compared specialist care leg orthoses to UC orthoses (control condition at baseline) in terms of personal goal attainment, walking ability outcomes and satisfaction. The patient population consisted of adults with NMD experiencing walking problems due to lower extremity muscle weakness. We hypothesized that specialist care leg orthoses would improve treatment outcomes compared to UC orthoses |
| Methods | | | |  |
| Study design | 4 | Present key elements of study design early in the paper | 5-6 |  |
| Setting | 5 | Describe the setting, locations, and relevant dates, including periods of recruitment, exposure, follow-up, and data collection | 5-6 | In this observational study, we retrospectively analysed data that were collected between October 2011 and March 2021 during orthotic care delivery in our university hospital outpatient polio and orthosis expertise rehabilitation clinic in Amsterdam, the Netherlands |
| Participants | 6 | (*a*) *Cohort study*—Give the eligibility criteria, and the sources and methods of selection of participants. Describe methods of follow-up  *Case-control study*—Give the eligibility criteria, and the sources and methods of case ascertainment and control selection. Give the rationale for the choice of cases and controls  *Cross-sectional study*—Give the eligibility criteria, and the sources and methods of selection of participants | 6 | On March 29, 2021 we searched our orthotic database to identify adults with NMD who had been assessed in our gait lab for gait problems. Eligibility criteria were: (1) minimum age of 18 years; (2) weakness of the calf muscles (i.e. Medical Research Council (MRC) scale [36] score < 5 or not being able to make three single heel-rises on one leg) and/or quadriceps muscles (i.e. MRC score < 5); (3) provision of their first specialist care orthosis; and (4) currently wearing a UC orthosis. We excluded patients for whom no follow-up data were available concerning evaluation of their specialist care orthosis. |
|  |  | (*b*) *Cohort study*—For matched studies, give matching criteria and number of exposed and unexposed  *Case-control study*—For matched studies, give matching criteria and the number of controls per case |  |  |
| Variables | 7 | Clearly define all outcomes, exposures, predictors, potential confounders, and effect modifiers. Give diagnostic criteria, if applicable | 8-10 | For the specification of outcomes, see pages 6-7. |
| Data sources/ measurement | 8* | For each variable of interest, give sources of data and details of methods of assessment (measurement). Describe comparability of assessment methods if there is more than one group | *8-10* |  |
| Bias | 9 | Describe any efforts to address potential sources of bias | N/A |  |
| Study size | 10 | Explain how the study size was arrived at | 10 | We identified 140 adults with NMD in the orthotic laboratory database who used a UC orthosis at baseline and were provided a specialist care orthosis during clinical care for walking problems due to calf muscle weakness and/or quadriceps weakness. Thereafter, 76 subjects were excluded as no follow-up measurements were (yet) available with their specialist care orthosis (Figure 1), leaving 64 subjects (35 males) with a mean (SD) age of 61 (12) years. Subjects were predominantly diagnosed with poliomyelitis (94%). |

Continued on next page

| Quantitative variables | 11 | Explain how quantitative variables were handled in the analyses. If applicable, describe which groupings were chosen and why | 10 | SPSS version 26 (IBM SPSS, Chicago, Illinois, USA) was used for all statistical analyses and significance levels were set at p<0.05. Subjects’ demographic (e.g. gender, age) and clinical characteristics (e.g. diagnosis, leg muscle strength) were presented using descriptive statistics. Differences between specialist care and UC orthoses were analysed with paired samples t-tests for continuous outcomes (walking speed and net energy cost). When data were not normally distributed and for ordinal data (GAS scores, perceived walking ability outcomes), Wilcoxon signed-rank tests were used. In addition, as working mechanisms of orthoses differ, effects in subgroups of specialist care orthosis types (AFO, SC-KAFO, locked KAFO) on walking speed, net energy cost and perceived walking ability were separately explored with paired samples t-tests or Wilcoxon signed-rank tests. |
| --- | --- | --- | --- | --- |
| Statistical methods | 12 | (*a*) Describe all statistical methods, including those used to control for confounding | 10 | Confounding N/A. |
|  |  | (*b*) Describe any methods used to examine subgroups and interactions | 10 | SPSS version 26 (IBM SPSS, Chicago, Illinois, USA) was used for all statistical analyses and significance levels were set at p<0.05. Subjects’ demographic (e.g. gender, age) and clinical characteristics (e.g. diagnosis, leg muscle strength) were presented using descriptive statistics. Differences between specialist care and UC orthoses were analysed with paired samples t-tests for continuous outcomes (walking speed and net energy cost). When data were not normally distributed and for ordinal data (GAS scores, perceived walking ability outcomes), Wilcoxon signed-rank tests were used. In addition, as working mechanisms of orthoses differ, effects in subgroups of specialist care orthosis types (AFO, SC-KAFO, locked KAFO) on walking speed, net energy cost and perceived walking ability were separately explored with paired samples t-tests or Wilcoxon signed-rank tests. |
|  |  | (*c*) Explain how missing data were addressed | N/A | Reasons for missing data will be reported in results section |
|  |  | (*d*) *Cohort study*—If applicable, explain how loss to follow-up was addressed  *Case-control study*—If applicable, explain how matching of cases and controls was addressed  *Cross-sectional study*—If applicable, describe analytical methods taking account of sampling strategy | N/A |  |
|  |  | (*e*) Describe any sensitivity analyses | N/A |  |
| Results | | | | |
| Participants | 13* | (a) Report numbers of individuals at each stage of study—eg numbers potentially eligible, examined for eligibility, confirmed eligible, included in the study, completing follow-up, and analysed | 10 | See Figure 1 |
|  |  | (b) Give reasons for non-participation at each stage | 10 | See Figure 1 |
|  |  | (c) Consider use of a flow diagram | 10 | See Figure 1 |
| Descriptive data | 14* | (a) Give characteristics of study participants (eg demographic, clinical, social) and information on exposures and potential confounders | 10-12 | See Figure 2 |
|  |  | (b) Indicate number of participants with missing data for each variable of interest | 12-16 | Indicated in results section per outcome measure |
|  |  | (c) *Cohort study*—Summarise follow-up time (eg, average and total amount) | 11 |  |
| Outcome data | 15* | *Cohort study*—Report numbers of outcome events or summary measures over time | *12-16* |  |
|  |  | *Case-control study—*Report numbers in each exposure category, or summary measures of exposure |  |  |
|  |  | *Cross-sectional study—*Report numbers of outcome events or summary measures |  |  |
| Main results | 16 | (*a*) Give unadjusted estimates and, if applicable, confounder-adjusted estimates and their precision (eg, 95% confidence interval). Make clear which confounders were adjusted for and why they were included | N/A |  |
|  |  | (*b*) Report category boundaries when continuous variables were categorized | N/A |  |
|  |  | (*c*) If relevant, consider translating estimates of relative risk into absolute risk for a meaningful time period | N/A |  |

Continued on next page

| Other analyses | 17 | Report other analyses done—eg analyses of subgroups and interactions, and sensitivity analyses | 12-16 | Reported per outcome measure |
| --- | --- | --- | --- | --- |
| Discussion | | | | |
| Key results | 18 | Summarise key results with reference to study objectives | 16 | Our findings indicate that guideline-based orthotic care within a multidisciplinary expertise setting leads to the attainment of personal goals by a majority of adults with NMD who use a leg orthosis for walking problems. Perceived walking ability also improves compared to usual orthotic care. While no overall effects on walking speed or net walking energy cost were found, the specialist care AFO group showed a significant and clinically relevant reduction in net energy cost. |
| Limitations | 19 | Discuss limitations of the study, taking into account sources of potential bias or imprecision. Discuss both direction and magnitude of any potential bias | 18 | A major strength of our study is that it shows the benefits of specialist care orthoses as provided in actual clinical practice to the largest cohort of patients with NMD so far. Nevertheless, several limitations need to be considered. First, the collection of data from actual clinical practice has led to missing data of subjects across outcomes. Second, follow-up measurements were performed after 3 months of specialist care orthosis use, whereas some UC orthoses had been in use for 2 years or more. Consequently, the effectiveness of UC orthoses at baseline might have declined due to orthosis wear and/or NMD progression. Finally, occasional later adjustments to the specialist care orthoses to improve functioning were based on the follow-up measurement, perhaps suggesting that ultimate effectivity might have been underestimated. In future research, treatment outcomes should be compared to a control group receiving usual orthotic care, preferably with randomization of interventions, allowing for a more accurate determination of the benefit of specialist orthotic care over usual care. |
| Interpretation | 20 | Give a cautious overall interpretation of results considering objectives, limitations, multiplicity of analyses, results from similar studies, and other relevant evidence | 12-15 |  |
| Generalisability | 21 | Discuss the generalisability (external validity) of the study results | 12-15 |  |
| Other information | |  | | |
| Funding | 22 | Give the source of funding and the role of the funders for the present study and, if applicable, for the original study on which the present article is based | - | Additional information submission article |

*Give information separately for cases and controls in case-control studies and, if applicable, for exposed and unexposed groups in cohort and cross-sectional studies.

**Note:** An Explanation and Elaboration article discusses each checklist item and gives methodological background and published examples of transparent reporting. The STROBE checklist is best used in conjunction with this article (freely available on the Web sites of PLoS Medicine at http://www.plosmedicine.org/, Annals of Internal Medicine at http://www.annals.org/, and Epidemiology at http://www.epidem.com/). Information on the STROBE Initiative is available at www.strobe-statement.org.
